# *dock11* Knockdown in Zebrafish Disrupts Embryogenesis: Insights Into the Genetic Causes of Early Pregnancy Loss

**DOI:** 10.1111/jcmm.71017

**Published:** 2026-01-08

**Authors:** Chang Liu, Meng Wang, Feng Chen, Mei Chen, Yonghua Yao, Wei Huang

**Affiliations:** ^1^ Department of Obstetrics and Gynecology West China Second University Hospital of Sichuan University Chengdu Sichuan China; ^2^ Key Laboratory of Birth Defects and Related Diseases of Women and Children of Ministry of Education Chengdu China; ^3^ NHC Key Laboratory of Chronobiology of Sichuan University Chengdu China; ^4^ West China Institutes for Women and Children's Health, West China Second University Hospital SCU‐CUHK Joint Laboratory for Reproductive Medicine Sichuan China; ^5^ Department of Operating Room Nursing West China Second University Hospital of Sichuan University Chengdu Sichuan People's Republic of China

**Keywords:** dedicator of cytokinesis 11, early embryogenesis, early pregnancy loss, zebrafish

Dear Editor,

Recurrent pregnancy loss (RPL), affecting approximately 5% of couples worldwide, represents a major challenge in reproductive medicine and causes psychological distress [1]. While embryonic chromosomal errors account for 40%–65% of early pregnancy losses, a substantial proportion of cases remain unexplained despite extensive clinical evaluation [2]. This diagnostic gap is further highlighted by the observation that pregnancy losses still occur even after the transfer of euploid embryos following preimplantation genetic testing for aneuploidy (PGT‐A) in assisted reproduction [3]. This clinical dilemma underscores a critical gap in our understanding of the molecular pathogenesis of early pregnancy loss, particularly the role of embryonic‐intrinsic factors [2, 3, 4]. While existing research has largely centered on deficits in implantation and placental development, the critical window of early embryogenesis—a period governed by the embryo's autonomous developmental program and fundamental to embryonic survival—has received comparatively less attention [5, 6, 7].

Our previous multi‐omics analysis of chorionic villi from euploid pregnancy‐loss patients revealed epigenetic silencing of *DOCK11 (dedicator of cytokinesis 11)* and its consequent transcriptional downregulation in extra‐embryonic tissues, implicating DOCK11 as a potential contributor to pregnancy failure (our unpublished data). This finding prompted us to investigate the potential intrinsic role of DOCK11 within the embryo proper.

To functionally validate the role of DOCK11 in early embryogenesis, we turned to the zebrafish model. This model is uniquely suited for such an investigation, as its external development and optical transparency enable direct visualization of embryogenesis while being free from the confounding influences of the maternal uterine environment and placental function. Morpholino (MO)‐mediated knockdown of *dock11* was confirmed via a significant reduction in its mRNA levels (Figure 1A). *Dock11*‐knockdown embryos exhibited markedly compromised viability, with significantly reduced hatching rates and elevated embryonic mortality compared to wild‐type (WT) controls (Figure 1B,C). Detailed morphological assessment revealed a spectrum of severe developmental defects, including pronounced axial curvature, a high incidence of malformations, and reduced overall body length (Figure 1D). To determine the impact on early patterning, we further performed whole‐mount in situ hybridization. Although the spatial domains of key lineage markers—including *gsc* and *chd* (dorsal mesoderm, assessed at 5 hpf), *bmp4* and *eve1* (ventral mesoderm, 5 hpf), *ntl* (axial mesoderm, assessed 8 hpf), *sox17* (endoderm, 8 hpf), and *gata2a* (ectoderm, 8 hpf) —remained largely unaltered in *dock11* MO embryos compared to WT embryos, their expression levels were markedly altered: the expression of *chd* was significantly increased, while that of *gsc*, *eve*1, *sox*17, and *gata2a* was decreased. Changes in *bmp4* and *ntl* were not significant (Figure 1E). Collectively, these findings establish that *dock11* is essential for normal embryogenesis and germ layer formation in zebrafish.

**FIGURE 1:**
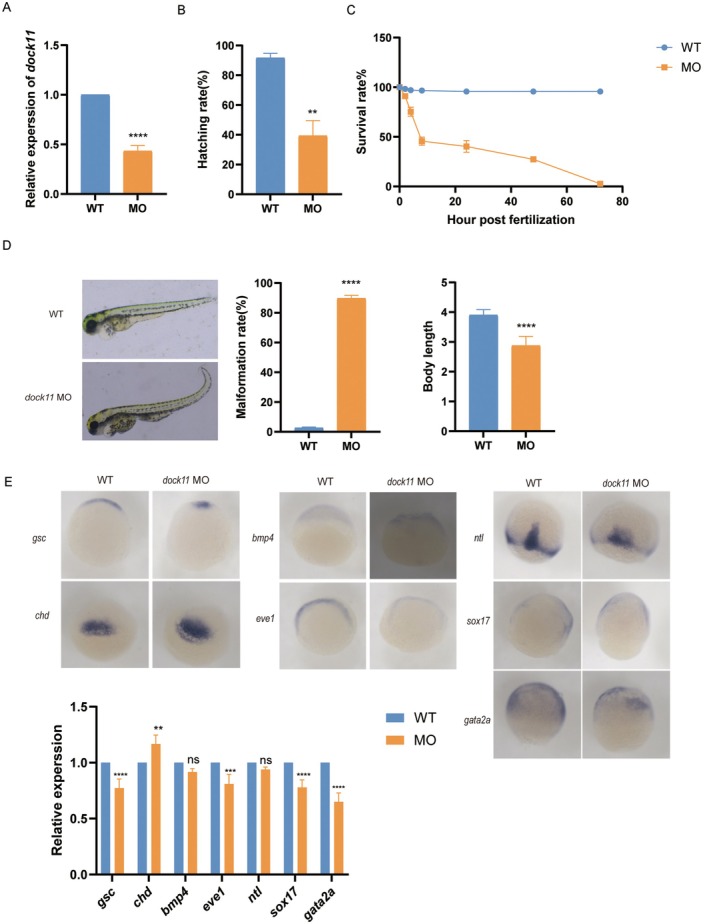
*dock11* affects development of zebrafish embryos. (A) The expression of *dock11* was determined by RT‐qPCR in wild‐type (WT) and morpholino (MO). mRNA levels were normalised to *gapdh* and are expressed relative to the WT control (*n* = 3). (B) Column chart shows the hatching rate of zebrafish. (C) Line chart represents the survival curve of zebrafish, with circular dotted line representing WT and square solid line indicating MO (*n* = 50). (D) Column graphs show the malformation rate (*p* < 0.0001) and body length (*p* < 0.0001) of zebrafish, respectively (*n* = 50). (E) Whole‐mount in situ hybridization of germ layer markers in WT and *dock11* MO zebrafish embryos. Column charts display the relative expression levels quantified from the signal intensity. Genes assessed at 5 hpf include *gsc*, *chd*, *bmp4*, and *eve1*; those assessed at 8 hpf include *nt1*, *sox17*, and *gata2a*. Data are presented as mean ± SD. *p < 0.05; **p < 0.01; ***p < 0.001, ****p < 0.0001.

Having established that the loss of *dock11* disrupts early embryonic development in zebrafish, we next sought to delineate the underlying molecular mechanisms. We first characterised the spatiotemporal expression profile of *dock11*, finding its mRNA to be dynamically expressed, with a pronounced peak at 5 h post‐fertilisation (hpf, Figure 2A)—a stage immediately preceding the onset of gastrulation that is critical for establishing the molecular patterns underlying subsequent cell fate determination. We therefore conducted transcriptomic profiling at this critical stage. RNA‐seq analysis revealed extensive dysregulation in *dock11* morphants, with 796 upregulated and 243 downregulated (Figure 2B,C, Table S1). Kyoto Encyclopedia of Genes and Genomes (KEGG) pathway analysis of these differentially expressed genes revealed enrichment in pathways related to embryonic development, including VEGF signalling, tight junction assembly, Hippo and MAPK signalling pathways (Figure 2D, Table S2). Furthermore, pathways governing cell fate, such as apoptosis, p53 signalling, ferroptosis, and cellular senescence, were prominently enriched, as were metabolic regulators like PPAR and AMPK signalling, and mRNA surveillance mechanisms (Figure 2D, Table S2). Gene ontology (GO) analysis of cellular component highlighted structures essential for tissue integrity and morphogenesis, including the extracellular matrix (ECM), endoplasmic reticulum membrane, ion‐channel complexes, the basolateral plasma membrane, and cell–cell junctions. Enrichment was also observed for cytoskeletal elements and vesicle‐related complexes (Figure 2E, Table S3). These results strongly suggest that *dock11* regulates a broad transcriptional network essential for cell adhesion, intra‐cellular signalling, and the maintenance of lineage integrity during early embryogenesis.

**FIGURE 2:**
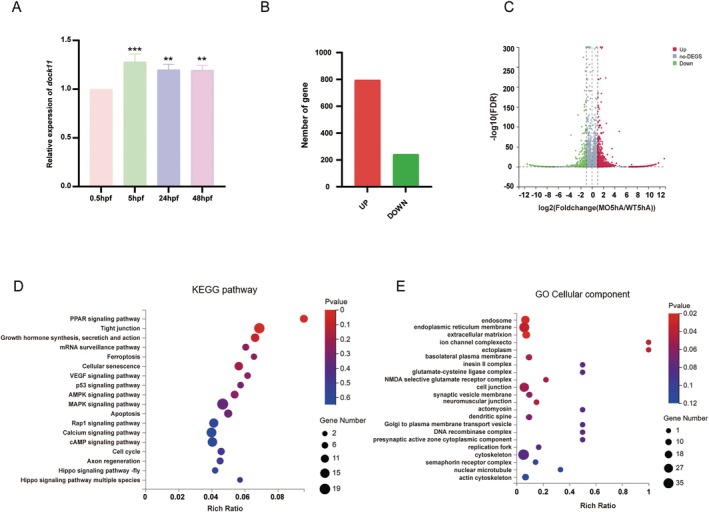
*dock11* regulates early transcriptional programs in zebrafish embryogenesis. (A) Column graphs show the mRNA expression levels of *dock11* in zebrafish embryos at 0.5 *hpf*, 5 *hpf*, 24 *hpf*, and 48 *hpf* (*n* = 3). (B) Column graphs show the number of DEGs related to *dock11*‐knockdown at 5 hpf zebrafish embryos. (C) Volcano plot shows the DEGs associated with *dock11* mRNA downregulation in the MO group compared with the WT group. (D) The bubble chart shows the representative KEGG pathways of DEGs associated with *dock11* mRNA downregulation. The *p*‐value was determined using a hypergeometric test. (E) Bubble chart shows the representative biological process GO terms enriched by DEGs related to the *dock11* mRNA downregulation. The *p*‐value was determined using a hypergeometric test. *p < 0.05; **p < 0.01; ***p < 0.001.

It is noteworthy that while previous studies using *dock11* knockout models in mice and zebrafish reported no embryonic lethality but rather focused on immune and hematopoietic phenotypes [8, 9], our study focuses on the role of *dock11* during early embryogenesis.

In conclusion, our integrated findings suggest the importance of embryonic‐intrinsic factors in pregnancy loss. We demonstrate that *dock11* is essential for body patterning and germ layer specification in the early vertebrate embryo. This work thus expands our understanding of the genetic aetiology of early pregnancy loss and establishes DOCK11 dysfunction as a previously unappreciated mechanistic contributor to the aetiology of early pregnancy loss.

## Author Contributions

Wei Huang: Conceptualised, designed, and supervised the research, revised the manuscript, and handled the submission. Chang Liu and Meng Wang: Performed the experiments, collected and analysed the data, and drafted the original manuscript. Feng Chen, Mei Chen, and Yonghua Yao: Assisted with experiments.

## Funding

This work was supported by National Key Research and Development Program of China, 2023YFC2705502. Natural Science Foundation of Sichuan province, 2025ZNSFSC0742. We acknowledge support from the National Key R&D Program of China (2023YFC2705502) and the General Program of Sichuan Provincial Natural Science Foundation (2025ZNSFSC0742).

## Ethics Statement

The study was approved by the Ethics Committee of West China Second University Hospital of Sichuan University (Approval No. 2025–51) and was approved by the Ethics Committee of Animal Experiments of Sichuan University (Approval No. 2025214). All participants provided written informed consent prior to their inclusion in the study.

## Consent

All authors reviewed the final manuscript and consented to its publication.

## Conflicts of Interest

The authors declare no conflicts of interest.

## Supporting information


**Table S1:** List of Differentially Expressed Genes from RNA‐seq Analysis of *dock11* Morphants.

**Table S2:** List of KEGG Pathway Enrichment Analysis for Differentially Expressed Genes from RNA‐seq of *dock11* Morphants.

**Table S3:** List of GO Enrichment Analysis for Differentially Expressed Genes from RNA‐seq of *dock11* Morphants.

**Table S4:** Sequences of primers used for quantitative real‐time PCR.

## Data Availability

The data that support the findings of this study are available from the corresponding author upon reasonable request.
