# Supplementary material for: dock11 Knockdown in Zebrafish Disrupts Embryogenesis: Insights Into the Genetic Causes of Early Pregnancy Loss
Source: J Cell Mol Med. 2026 Jan 8;30(1):e71017. doi: 10.1111/jcmm.71017 (PMC12780853; doi:10.1111/jcmm.71017)
Supplement: Supplementary file 4 — Table S4: Sequences of primers used for quantitative real‐time PCR. [file JCMM-30-e71017-s002.docx]

**Table S4** **Sequences of primers used for quantitative real-time PCR**

| Gene | Forward Primer (5' to 3') | Reverse Primer (5' to 3') |
| --- | --- | --- |
| *dock11* | TCGGGAGATTTCCGGATGCT | ATCATCTCTTACCTCCTCCCCC |
| *gapdh* | CGTGGCCATCAATGACCCAT | CGCCTTCTGCCTTAACCTCA |
